# Improvement of maternal Aboriginality in NSW birth data

**DOI:** 10.1186/1471-2288-12-8

**Published:** 2012-01-30

**Authors:** Fenglian Xu, Elizabeth A Sullivan, Richard C Madden, Deborah Black, Lisa R Jackson Pulver

**Affiliations:** 1 PRERU, University of New South Wales, Randwick, NSW 2031, Australia; 2 Faculty of Health Sciences, Sydney University, Lidcombe NSW 1825, Australia; 3 Muru Marri Indigenous Health Unit, School of Public Health & Community Medicine, University of New South Wales, NSW 2052, Australia

**Keywords:** Birth, Aboriginality, data, Australia

## Abstract

**Background:**

The Indigenous population of Australia was estimated as 2.5% and under-reported. The aim of this study is to improve statistical ascertainment of Aboriginal women giving birth in New South Wales.

**Methods:**

This study was based on linked birth data from the Midwives Data Collection (MDC) and the Registry of Births Deaths and Marriages (RBDM) of New South Wales (NSW). Data linkage was performed by the Centre for Health Record Linkage (CHeReL) for births in NSW for the period January 2001 to December 2005. The accuracy of maternal Aboriginal status in the MDC and RBDM was assessed by consistency, sensitivity and specificity. A new statistical variable, ASV, or Aboriginal Statistical Variable, was constructed based on Indigenous identification in both datasets. The ASV was assessed by comparing numbers and percentages of births to Aboriginal mothers with the estimates by capture-recapture analysis.

**Results:**

Maternal Aboriginal status was under-ascertained in both the MDC and RBDM. The ASV significantly increased ascertainment of Aboriginal women giving birth and decreased the number of missing cases. The proportion of births to Aboriginal mothers in the non-registered birth group was significantly higher than in the registered group.

**Conclusions:**

Linking birth data collections is a feasible method to improve the statistical ascertainment of Aboriginal women giving birth in NSW. This has ramifications for the ascertainment of babies of Aboriginal mothers and the targeting of appropriate services in pregnancy and early childhood.

## Background

The Indigenous population of Australia was estimated as 2.5% (517,200) of the Australian population as of 30 June 2006 [1,2]. New South Wales (NSW) had the largest proportion of the Indigenous estimated resident population (29-30%) in Australia [1-3]. An estimated 95% of the NSW Indigenous population were of Aboriginal origin, 3% were of Torres Strait Islander origin and the remaining 2% were of both Aboriginal and Torres Strait Islander origin [1].

In a report by the Australian Institute of Health and Welfare (AIHW), 35,264 women who identified as being of Indigenous origin gave birth to 35,682 babies (3.6% of all babies) between 2001 and 2004 [4]. The report is based on data from the National Perinatal Data Collection (NPDC) which is derived from an extract of the Midwives Data Collections (MDC) in each state and territory in Australia [4].

However, it was found that maternal Indigenous status was under-reported in the New South Wales (NSW) MDC and the Registry of Birth Deaths and Marriages (RBDM) birth [5-9]. In the annual NSW Mothers and Babies published by the AIHW, about only 65.5-69.3% of Indigenous mothers were identified as Indigenous status in the MDC from 2002 to 2005 [5,10,11]. The Australian Bureau of Statistics (ABS) estimated that only 89.3% of Indigenous mothers were identified as Indigenous in the NSW RBDM from 2002 to 2006 [8].

To adjust for under-enumeration, capture-recapture methods were used to estimate the number of Indigenous mothers and babies [6,7]. The capture-recapture method was derived from techniques used in wildlife science to estimate the size of animal populations. The method was applied to epidemiology and became a useful tool to reduce under-estimation of cases or study population [12]. It was used to estimate the population with hidden natures such as alcohol-related problems, mental illness, human immunodeficiency virus infections and diabetes [13-16]. It also was used to evaluate completeness of cancer surveillance and cerebral palsy registry [17,18].

However, the summary numbers calculated by the capture-recapture method were not on an individual level and could not be used for some data analyses such as multivariate regression analysis. An improved statistical variable of Aboriginality is needed to more accurately reflect the Aboriginal population in NSW.

The under-identification of Indigenous patients was also apparent in other states and territories in Australia [19]. A report by AIHW based on hospital separation data from Queensland, South Australia, Western Australia and the Northern Territory showed that only 89% of Indigenous patients were identified in public hospital admission records in 2007-08 [19]. An ABS Census showed that the Indigenous population at the time of the 2006 Census was 454,799, while the Census Post Enumeration Survey (PES) estimate was 513,977 people. The difference of 59,178 represents 11.5% of the PES estimate. However, the net undercount rate for the total population was only 2.7% [1].

The aim of this study is to improve the statistical ascertainment of Aboriginal women giving birth in New South Wales. The method can be a reference for improving the data quality of Indigenous populations in other states and territories in Australia. It can also be used for quality improvement of other variables which are available in different data collections by data linkage.

## Methods

### Data sources

This study is based on linked population data of births (MDC-RBDM) from January 2001 to December 2005 in New South Wales. The MDC was recorded by midwives and other health care professionals. The scope of the MDC was all live and stillbirths of at least 400 grams birthweight or at least 20 weeks gestation in NSW. Comparatively, the RBDM was recorded by the parents of the child and lodged with the Registrar of Births Deaths and Marriages. It covered all births registered in New South Wales and included information on demographic factors and some pregnancy outcomes.

The Indigenous status in the MDC only includes that relating to the mother. The Indigenous status in the RBDM birth includes that of both the mother and the father.

The values of maternal Indigenous status in both birth data collections include: (1) Aboriginal, (2) Torres Strait Islander, (3) both Aboriginal and Torres Strait Islander and (4) neither Aboriginal nor Torres Strait Islander.

There are few births in NSW reported by Torres Strait Islander mothers, so the combined value of Indigenous status was used in the study.

### Data linkage

Centre for Health Record Linkage (CHeReL) performed the data linkage using probabilistic record linkage methods and choiceMaker software. At the completion of the process, each record in the Master Linkage Key was assigned a record identification number and a Master Linkage Key person ID (Person Project Number (PPN)) to allow linked records for the same individual to be identified and extracted. Checked by 1,000 person IDs selected at random, the false positive rate of the linkage was 0.3% and false negative < 0.1%.

### Definition of Indigenous status

Indigenous status refers to Aboriginal and Torres Strait Islander origin. Maternal Indigenous status refers to women who have given birth and identify themselves to be of Aboriginal and Torres Strait Islander origin [5].

### Assessment of the accuracy of Indigenous status in mothers who reported to be Indigenous

The accuracy of maternal Indigenous status in the MDC and RBDM birth can be assessed for consistency, sensitivity and specificity. For the purposes of this study, the sensitivity and specificity are based on the assumption that the Indigenous status reported in the MDC and RBDM are correct. So the Indigenous status in the MDC can work as the 'standard' for the calculation of the sensitivity and specificity in the RBDM birth and vice versa. Because the sensitivity and specificity are calculated based on assumptions rather than 'gold standard', the sensitivity in this paper is a 'relative sensitivity' and the specificity is a 'relative specificity'.

The formulas for calculating consistency, sensitivity and specificity are as below. Table 1 illustrates the letters in the formulas.

**Table 1:** The illustration for calculating consistency, sensitivity and specificity


	**RBDM**			

	**Values**	**Indigenous**	**Non-Indigenous**	**Total**

**MDC**	Indigenous	A	B	A+B
	
	Non-Indigenous	C	D	C+D
	
	Total	A+C	B+D	T

Consistency refers to the probability of the same identification of maternal Aboriginality between MDC and RBDM in the Indigenous population (Consistency (%) = (A+D)/T*100). Sensitivity refers to the probability that maternal Aboriginality is identified in Indigenous population (true positive). Sensitivity of MDC (%) is A/(A+C)*100; sensitivity of RBDM (%) is A/(A+B)*100. Specificity refers to the probability that maternal non-Aboriginality is identified in the non-Indigenous population. Specificity of MDC (%) is D/(B+D)*100, and specificity of RBDM (%) is D/(C+D)*100. Completeness refers to the extent to which individual variables have non-missing records.

### The statistical Aboriginal variable (ASV)

An improved variable, ASV was created by linking the MDC and RBDM birth. The ASV is based on the following assumptions:

1. Mothers self-reported as Indigenous in either the MDC or RBDM are recorded as Indigenous in ASV.

2. Mothers self-reported as non-Indigenous in both the MDC and RBDM are recorded as non-Indigenous.

3. Mothers self-reported as non-Indigenous in the MDC are recorded as non-Indigenous when the value in the RBDM birth is not stated, and vice versa.

4. Mothers, where the Indigenous status is not stated in the MDC or the RBDM, are classified as missing.

The ASV is an aggregate variable transformed through the linkage of the MDC and RBDM.

### The assessment of ASV

Using capture-recapture analysis, an estimate of the population prevalence of births with Aboriginal and/or Torres Strait Islander mothers can be calculated. The conditions for using the capture-recapture method are met in this study [7,20]. The ASV can be assessed by comparing the numbers and percentages of births to Aboriginal mothers with the estimates by capture-recapture analysis. The population of births with Aboriginal mothers (N) can be calculated by the formula below [16,20,21]:

$$\text{N}=\frac{\left(n_{1}+1\right)\left(n_{2}+1\right)}{\left(n_{12}+1\right)}-1$$

Where n_1 _is the number of births to Aboriginal mothers identified by the MDC; n_2 _is the number of births to Aboriginal mothers identified by the RBDM birth; and n_12 _is the number of births to Aboriginal mothers identified by both the MDC and RBDM birth.

Approximate unbiased estimate of the variance of N [16,20,21]:

$$\text{Var}\left(\text{N}\right)=\left[\left({\text{n}}_{1}+1\right)\left({\text{n}}_{2}+1\right)\;\left({\text{n}}_{1}-{\text{n}}_{12}\right)\left({\text{n}}_{2}-{\text{n}}_{12}\right)\right]/\left[{\left({\text{n}}_{12}+1\right)}^{2}\;\left({\text{n}}_{12}+2\right)\right]$$

The 95% confidence intervals (95%CI) of N are:

$$95\%\text{CI}=\text{N}\pm 1.96*\;\sqrt{Var\left(N\right)}$$

The estimates of Aboriginal mothers by capture-recapture are assumed to represent the true number or rate of Aboriginal mothers. The maternal Indigenous status was under-reported in the MDC and RBDM and the numbers of Aboriginal mothers were significantly below the estimates calculated by the capture-recapture method. If the ASV can improve the under-reported numbers, it should be higher than the reported number on the MDC and RBDM birth and closer to the estimates by capture-recapture analysis; the closer, the better. If there is no significant difference between the results of the ASV and the estimates from capture-recapture analysis, the ASV is considered to be improved to an optimal level and can represent the true number of Aboriginal mothers.

The chi-square test and 95% confidence interval were used to assess the differences between the percentages analysed by the ASV and the estimates calculated by the capture-recapture method.

### Ethics

The linkage study was approved by the Aboriginal Health and Medical Research Council, NSW Population & Health Services Research Ethics Committee and the Human Research Ethics Committees of The University of Sydney and the University of New South Wales. Identifying information such as name, address, date of birth and gender obtained from the MDC baby and RBDM birth datasets was included in the Master Linkage Key which was constructed by the (CHeReL).

## Results

There were a total of 440,994 birth records from the MDC and RBDM between January 2001 and December 2005 in NSW. There were 434,467 birth records in the MDC and 404,734 birth records in the RBDM birth. Theoretically, all births in the NSW MDC can be linked with those from the NSW RBDM birth. However, some birth records cannot be linked. There were 398,207 records which could be linked between the MDC and RBDM births with a linkage rate of 91.65% in the MDC and 98.39% in the RBDM birth.

There were some babies who had more than one record in the data collections, so the duplicate records of 677 (one in the linked database, 45 in the non-linked MDC and 631 in the non-linked RBDM) were excluded from the analysis. Eleven records did not state maternal Indigenous status in either the MDC or the RBDM birth and were excluded from the analysis.

Table 2 is based on linked records of the MDC and RBDM between 2001 and 2005. It shows the consistency of maternal Indigenous status between the MDC and RBDM, and sensitivity and specificity of maternal Indigenous status in the MDC and RBDM.

**Table 2:** Accuracy of maternal Indigenous status in the MDC and RBDM birth 2001-2005, NSW


		**Consistency**		**MDC**		**RBDM birth**	
**Year**							
	**n**	**n_1_**	**%**	**Sensitivity**	**Specificity**	**Sensitivity**	**Specificity**

2001	80,027	79,191	98.96	65.47	99.77	87.20	99.17

2002	78,338	77,495	98.92	65.30	99.74	85.90	99.16

2003	77,756	76,966	98.98	66.69	99.75	86.59	99.21

2004	79,389	78,555	98.95	67.15	99.72	85.32	99.21

2005	74,631	73,974	99.12	69.33	99.76	85.88	99.35

Total	390,141	386,181	98.98	66.70	99.75	86.18	99.22

Missing records total 8,066, accounting for 2.0%.

n: number of linked records between MDC and RBDM

n_1_: consistent number between MDC and RBDM

The mother's Indigenous statuses were highly consistent between the MDC and RBDM. However, the relative sensitivities in both MDC and RBDM were low in both the MDC and RBDM birth, and the sensitivities in the MDC were lower than the RBDM birth. At least one-third of Indigenous mothers were not identified in the MDC and one-seventh in the RBDM birth.

Table 3 is based on linked records of the MDC and RBDM birth. Estimates were computed using the capture-recapture method. For the linked births, the percentage of maternal Indigenous status was lower in the MDC (1.82%) than in the RBDM (2.31%) birth. This compares to the percentages of maternal Indigenous status from the ASV (2.57%) which were significantly higher than the MDC and RBDM birth alone. Compared with the estimates by the capture-recapture method (2.68%), the percentages of maternal Indigenous status from the ASV were very close to the estimates and were not significantly different to the estimates each year (p > 0.05). However, the overall percentage of maternal Indigenous status from the ASV was lower than the estimate by the capture-recapture method.

**Table 3:** Maternal Indigenous status in linked birth data (MDC and RBDM birth), 2001-2005, NSW


		**Maternal Indigenous status**					**Missing**		

**Sources**	**Year**	**n1**	**Indigenous**	**%**	**95%CI**		**n2**	**Missing records**	**%**

MDC	2001	80,931	1,489	1.84	1.75	1.93	80,972	41	0.05
	2002	81,303	1,497	1.84	1.75	1.93	81,348	45	0.06
	2003	81,131	1,472	1.81	1.72	1.90	8,1167	36	0.04
	2004	79,972	1,517	1.90	1.81	1.99	80,003	31	0.04
	2005	74,631	1,261	1.69	1.60	1.78	74,717	86	0.12

	Total	397,968	7,236	1.82	1.78	1.86	398,207	239	0.06

RBDM birth	2001	80,068	1,894	2.37	2.26	2.48	80,972	904	1.12
	2002	78,378	1,858	2.37	2.26	2.48	81,348	2,970	3.65
	2003	77,786	1,810	2.33	2.22	2.44	81,167	3,381	4.17
	2004	79,420	1,879	2.37	2.26	2.48	80,003	583	0.73
	2005	74,717	1,562	2.09	1.99	2.19	74,717	0	0.00

	Total	390,369	9,003	2.31	2.26	2.36	398,207	7,838	1.97

ASV	2001	80,972	2,143	2.65	2.54	2.76	80,972	0	0.00
	2002	81,343	2,143	2.63	2.52	2.74	81,348	5	0.01
	2003	81,161	2,075	2.56	2.45	2.67	81,167	6	0.01
	2004	80,003	2,135	2.67	2.56	2.78	80,003	0	0.00
	2005	74,717	1,740	2.33	2.22	2.44	74,717	0	0.00

	Total	398,196	10,236	2.57	2.52	2.62	398,207	11	0.00

Estimates by capture-recapture method	2001	80,027	2,172	2.71	2.68	2.75	80,972	945	1.17
	2002	78,338	2,161	2.76	2.72	2.79	81,348	3,010	3.70
	2003	77,756	2,090	2.69	2.66	2.72	81,167	3,411	4.20
	2004	79,389	2,201	2.77	2.74	2.81	80,003	614	0.77
	2005	74,631	1,819	2.44	2.41	2.47	74,717	86	0.12

	Total	390,141	10,443	2.68	2.64	2.71	398,207	8,066	2.03

n1 Records without 'missing' in Indigenous status

n2 Total linked records

Table 4 is based on linked and unlinked birth records. The ASV of maternal Indigenous statuses in the MDC (ASV in MDC) includes the values from the ASV in linked records and unlinked MDC records. Similarly, the maternal Indigenous statuses in RBDM birth (ASV in RBDM birth) include the values from ASV in linked records and unlinked RBDM records. The estimates by the capture-recapture method are based on all records in the MDC or RBDM birth.

**Table 4:** Improvement of maternal Indigenous status in MDC and RBDM birth respectively, 2001-2005, NSW


		**Maternal Indigenous status**					**Missing**		

**Sources**	**Year**	**n1**	**Indigenous**	**%**	**95%CI**		**n2**	**Missing records**	**%**

MDC	2001	85,809	2,138	2.49	2.39	2.59	85,855	46	0.05
	2002	85,954	2,183	2.54	2.43	2.65	86,003	49	0.06
	2003	86,366	2,189	2.53	2.43	2.63	86,406	40	0.05
	2004	85,584	2,333	2.73	2.62	2.84	85,616	32	0.04
	2005	90,491	2,506	2.77	2.66	2.88	90,587	96	0.11

	Total	434,204	11,349	2.61	2.56	2.66	434,467	263	0.06

ASV in MDC	2001	85,850	2,792	3.25	3.13	3.37	85,855	5	0.01
	2002	85,994	2,829	3.29	3.17	3.41	86,003	9	0.01
	2003	86,396	2,792	3.23	3.11	3.35	86,406	10	0.01
	2004	85,615	2,951	3.45	3.33	3.57	85,616	1	0.00
	2005	90,577	2,985	3.30	3.18	3.42	90,587	10	0.01

	Total	434,432	14,349	3.30	3.25	3.35	434,467	35	0.01

RBDM birth	2001	81,630	1,944	2.38	2.28	2.48	82,559	929	1.13
	2002	79,871	1,896	2.37	2.26	2.48	82,910	3,039	3.67
	2003	79,028	1,852	2.34	2.23	2.45	82,470	3,442	4.17
	2004	80,540	1,907	2.37	2.26	2.48	81,141	601	0.74
	2005	75,654	1,582	2.09	1.99	2.19	75,654	0	0

	Total	396,723	9,181	2.31	2.26	2.36	404,734	8,011	1.98

ASV in RBDM birth	2001	82,534	2,193	2.66	2.55	2.77	82,559	25	0.03
	2002	82,836	2,181	2.63	2.52	2.74	82,910	74	0.09
	2003	82,403	2,117	2.57	2.46	2.68	82,470	67	0.08
	2004	81,123	2,163	2.67	2.56	2.78	81,141	18	0.02
	2005	75,654	1,760	2.33	2.22	2.44	75,654	0	0

	Total	404,550	10,414	2.57	2.52	2.62	404,734	184	0.05

Estimates by capture-recapture method	2001	87,442	3,351	3.83	3.75	3.92			
	2002	87,565	3,415	3.90	3.81	3.99			
	2003	87,709	3,358	3.83	3.74	3.91			
	2004	86,754	3,528	4.07	3.98	4.15			
	2005	91,524	3,660	4.00	3.90	4.10			

	Total	440,994	17,312	3.93	3.84	4.01			

n1 Records without 'missing' in Indigenous status

n2 Total birth number

The percentages of maternal Indigenous status from the ASV in the MDC and the ASV in the RBDM birth were significantly higher than the original maternal Indigenous statuses in the MDC and RBDM birth respectively. The ASV in the MDC and RBDM birth also reduced the number of missing cases significantly (p < 0.05), especially for the ASV RBDM birth (except for 2005). The improvement in the MDC was more than the RBDM birth. However, the percentages of maternal Indigenous status from the ASV in the MDC and the ASV in the RBDM birth were still significantly lower than the estimates by the capture-recapture method.

Table 5 is based on all birth records including linked records (MDC-RBDM birth), not linked records in the MDC (MDC records only) and not linked records in the RBDM birth (RBDM birth records only). There were more births to Aboriginal women in the non-registered group than the registered group (about five times). However, the maternal Indigenous statuses were not significantly different in the linked registered group and the non-linked registered group.

**Table 5:** Maternal Aboriginality in registered and non-registered groups, 2001-2005, NSW


**Year**	**Birth records**	**n**	**Indigenous number**	**%**	**95% CI**	

2001	Registered^a^	80,972	2,143	2.65	2.54	2.76
	Registered^b^	1,562	50	3.20	2.33	4.07
	Non-registered	4,878	649	13.3	12.35	14.25
	
	Total	87,412	2,842	3.25	3.13	3.37

2002	Registered^a^	81,343	2,143	2.63	2.52	2.74
	Registered^b^	1,493	38	2.55	1.75	3.35
	Non-registered	4,651	686	14.75	13.73	15.77
	
	Total	87,487	2,867	3.28	3.16	3.4

2003	Registered^a^	81,161	2,075	2.56	2.45	2.67
	Registered^b^	1,242	42	3.38	2.37	4.39
	Non-registered	5,235	717	13.7	12.77	14.63
	
	Total	87,638	2,834	3.23	3.11	3.35

2004	Registered^a^	80,003	2,135	2.67	2.56	2.78
	Registered^b^	1,120	28	2.5	1.59	3.41
	Non-registered	5,612	816	14.54	13.62	15.46
	
	Total	86,735	2,979	3.43	3.31	3.55

2005	Registered^a^	74,717	1,740	2.33	2.22	2.44
	Registered^b^	937	20	2.13	1.21	3.05
	Non-registered	15,860	1,245	7.85	7.43	8.27
	
	Total	91,514	3,005	3.28	3.16	3.4

Total	Registered^a^	398,196	10,236	2.57	2.52	2.62
	Registered^b^	6,354	178	2.8	2.39	3.21
	Non-registered	36,236	4,113	11.35	11.02	11.68
	
	Total	440,786	14,527	3.3	3.25	3.35

Registered^a^: Linked birth records of MDC-RBDM.

Registered^b^: RBDM birth records which cannot be linked with MDC.

Non-registered: MDC birth records which cannot be linked with RBDM.

## Discussion

The Aboriginal status of women giving birth in NSW was under-reported in both the MDC and RBDM birth [5-9]. Table 3 shows the results of linked births, the births which had been registered. Table 4 shows the results of all births recorded in the MDC and RBDM. The absolute number of Aboriginal mothers identified in the MDC was less than the RBDM birth in linked data (Table 3) but more than the RBDM birth when unlinked births in the MDC and RBDM birth (Table 4) were included respectively. In the births that were registered, the babies born to Indigenous mothers in the MDC (7,236) were less than those in the RBDM birth (9,003) (Table 3). The sensitivity of the MDC (66.7%) was lower than that of the RBDM birth (86.2%). But if non-registered births were included in the MDC, the number of babies born to Indigenous mothers in the MDC (11,349) was more than in the RBDM birth (9,181) (Table 4) because Indigenous births are less likely to be registered. Though the data quality of Aboriginality in the RBDM was better than the MDC, many births of Indigenous mothers were not registered. Increasing the rate of birth registrations will be an effective way to improve the data quality of Indigenous status.

However, the under-ascertainment of maternal Aboriginal status can be improved significantly by creating the ASV through linkage of the MDC and RBDM birth. The ASV maximises ascertainment through accepting as valid Indigenous identification in both datasets. The linkage minimises missing values in both the MDC and RBDM birth. The ASV is more accurate and complete and is more suitable for use in advanced computation such as regression analysis.

The ASV improves the data quality at an individual record level rather than providing an estimation of the total number calculated by the capture-recapture method. As a result the ASV can be used for more analyses which are based on individual records. For example, the variable is not only used to describe the rate and distribution of the Indigenous population but can also be used as a independent factor to show the association with other dependent factors such as low birthweight or stillbirth. However, the estimation by the capture-recapture method is an overall number which cannot be used for factor analysis.

Interestingly, although the ASV improves ascertainment of Aboriginal women giving birth, it still under-enumerates estimates derived using a capture-recapture methodology. The impact of this limitation could be minimised by internal data linkage such as linking all of a mother's births to check the Indigenous identification. For example, if a mother had more than one birth, the missing or incorrect values of Aboriginality can be supplemented or confirmed with multiple birth records for that mother. But internal data linkage ideally involves a longer study period given that a mother may have more than one birth. The five-year study period was not long enough for internal linkage. This limitation may also be addressed through additional linkages of other data sources such as Medicare data collection, Admitted Patients Data Collection (APDC), the Emergency Department Data Collection (EDDC) or Register of Congenital Conditions (RCC). It is therefore possible to further improve the ascertainment of Aboriginality to an optimal level by linking the birth data with additional data collections of information on Aboriginality.

Indigenous population was under-estimated not only in New South Wales but also in other states and territories in Australia [19]. The ASV is an option to improve the quality of Indigenous status. On the other hand, data linkage provides an effective way to create or derive statistical variables besides the ASV. It improves the data quality of other variables such as a mother's country of birth and in broader areas such as the study of substance use in which some diagnoses in the APDC in specific perinatal periods can be confirmed by the Pharmaceutical Drugs of Addiction System (PHDAS).

The percentage of births to Aboriginal mothers in the registered birth group in 2005 was significantly lower than in the preceding four-year period, 2001 to 2004. This is because Aboriginal babies' registrations are more likely to be delayed [8]. For example, the MDC birth records in 2001 can be linked to the RBDM birth from 2001 to 2005, 2002 can be linked to 2002 to 2005, and so on. But the delayed registration records after 2005 were not available for the MDC records in 2005 to link.

The number of births between 2001 and 2005 in the MDC (Table 4) was marginally lower than reported by New South Wales Mothers and Babies [5,22]. This is because 46 duplicate records were excluded from this analysis. The number of births recorded between 2001 and 2005 in the RBDM was significantly lower (25,254) than that reported by the ABS [8]. This is because the year used is year of birth while the year in the ABS report refers to year of registration. Furthermore, the data sources were also different. The data used is from registration and the data for the ABS report is from registration and the Census [8].

The assumptions for using the capture-recapture method are described in Additional file 1[23-25].

All linkage studies have limitations. The sensitivity and specificity were calculated based on the assumption that the Indigenous status of women reported in the MDC and RBDM birth was the true and valid status, rather than a 'gold standard'. A validation study of these data would be needed to test this assumption. This is not possible as de-identified data made available from NSW Health is used. The sensitivity and specificity detail differences in the quality of the MDC and RBDM birth.

## Conclusion

Linkage of the birth data collections is a feasible method to improve the statistical ascertainment of Aboriginal women giving birth in NSW. Linkage results in enhanced ascertainment and the construction of a statistical Aboriginal variable. This can be applied to both of the birth collections and other population data. This has ramifications for the ascertainment of babies of Aboriginal mothers and the targeting of appropriate services in pregnancy care and early childhood. Increasing birth registration rate and additional data linkage may improve the ascertainment further.

## Competing interests

The authors declare that they have no competing interests.

## Authors' contributions

FX participated in study design, data analysis and paper writing. EAS participated in study design, coordination and paper writing. RCM conceived of the study, designed, coordinated and supervised the study. DAB participated in data analysis. LRJP oversaw the aspects of the analysis and reporting of information relating to Indigenous status. All authors read, revised the manuscript and approved the final version.

## Pre-publication history

The pre-publication history for this paper can be accessed here:

http://www.biomedcentral.com/1471-2288/12/8/prepub

## Supplementary Material

Additional file 1 **Assumptions for using the capture-recapture method**. Five assumptions were considered when using the estimates by the capture-recapture method. Click here for file
