# Supplementary material for: Improvement of maternal Aboriginality in NSW birth data
Source: BMC Med Res Methodol. 2012 Jan 30;12:8. doi: 10.1186/1471-2288-12-8 (PMC3314542; doi:10.1186/1471-2288-12-8)
Supplement: Additional file 1 — Assumptions for using the capture-recapture method. Five assumptions were considered when using the estimates by the capture-recapture method. [file 1471-2288-12-8-S1.DOCX]

# Additional file 1 (Improvement of maternal Aboriginality in NSW birth data)

The following assumptions should be considered when using the estimates by the capture–recapture method.

1. Closed-population

The methodology assumes that the population is closed, similar to a population in a fishpond[[23](#_ENREF_23)]. The same individual births must be present during the study period between birth and registration. In reality, no population is closed because of death and migration. So this assumption can only be satisfied to a limited degree. For the current study, the effect of the violation of the closed-population assumption was minimised because duration between birth and registration was very short and the population was sufficiently stable. Under the Births, Deaths and Marriages Registration Act 1995 all births in NSW are to be registered within 60 days[[24](#_ENREF_24)].

1. Homogeneity or equal catchability

The second assumption is capture homogeneity[[23](#_ENREF_23)]. In any single data source, each birth had the same probability of ascertainment (although any two sources may differ in this probability)[[23](#_ENREF_23)]. The MDC included all births in NSW and relied on the attending midwife or doctor to complete a notification form when a birth occurred. The form included a demographic item of Indigenous status with four options: 1. Aboriginal, 2. Torres Strait Islander, 3. Aboriginal and Torres Strait Islander and 4. None of the above[[25](#_ENREF_25)]. Women who self-identify as having Aboriginal and/or Torres Strait Islander heritage were recorded as an Indigenous person. Each mother in the MDC had an equal probability of being identified as Indigenous. The RBDM was based on the **Birth Registration Statement** (BRS) supplied by the parents and each parent had an equal probability of being reported as Indigenous.

1. Independence of notification sources

The third assumption is that identification by one source should not impact the chance of being identified by the other source [[12](#_ENREF_12), [18](#_ENREF_18)]. Positive dependence occurs when identification by one source increases the probability of being identified by another source. On the contrary, the dependence is negative when identification by one source decreases the probability of being identified by another source. Positive dependence under-estimates the population size, while negative dependence overestimates the population[[17](#_ENREF_17)]. The MDC consisted of medical records which were completed by midwives and other health care professionals. However, the RBDM was based on the report from parents. The two data collections were independent.

1. Correct matching

The fourth assumption is that the individuals in different sources were matched appropriately[[23](#_ENREF_23)]. The data linkage for this study used probabilistic record linkage methods and choiceMaker software. The true matching was maximised with a 0.3% false positive rate and less than 0.1% false negative rate.

1. Correct identification

The fifth assumption is that the identified cases are true cases[[20](#_ENREF_20)]. In the current study, it is assumed that all the Aboriginality identified in the data collections were correct. In fact, mothers were more likely to be incorrectly identified as non-Aboriginal than Aboriginal[[7](#_ENREF_7)]. The false-negative identification caused an under-estimate of the Aboriginal population[[7](#_ENREF_7)]. As a result, the true indigenous population may be larger than the current estimation.
